# Volatile and Nonvolatile Programmable Iontronic Memristor with Lithium Imbued TiO_*x*_ for Neuromorphic Computing Applications

**DOI:** 10.1021/acsnano.4c05137

**Published:** 2024-08-07

**Authors:** Rabiul Islam, Yu Shi, Gabriel Vinicius de Oliveira Silva, Manoj Sachdev, Guo-Xing Miao

**Affiliations:** † Department of Electrical and Computer Engineering, University of Waterloo, Waterloo N2L 3G1, Ontario, Canada; ‡ Institute for Quantum Computing, University of Waterloo, Waterloo N2L 3G1, Ontario, Canada

**Keywords:** artificial synapses, iontronics, Li-ion doped TiO_*x*_, neuromorphic computing, reservoir computing, volatile memristors

## Abstract

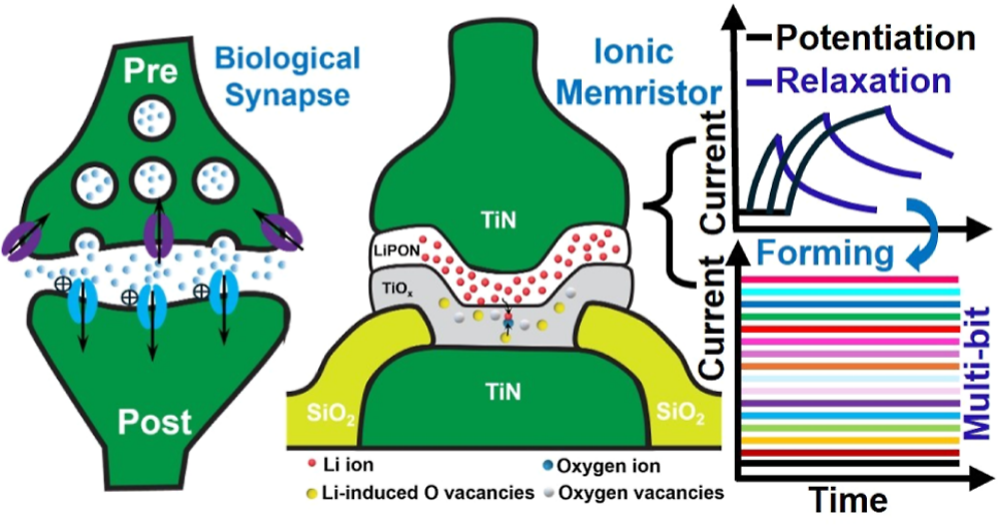

We demonstrate a lithium (Li) imbued TiO_*x*_ iontronic device that exhibits synapse-like short-term plasticity behavior without requiring a forming process beforehand or a compliance current during switching. A solid-state electrolyte lithium phosphorus oxynitride (LiPON) behaves as the ion source, and the embedding and releasing of Li ions inside the cathodic like TiO_*x*_ renders volatile conductance responses from the device and offers a natural platform for hardware simulating neuron functionalities. Besides, these devices possess high uniformity and great endurance as no conductive filaments are present. Different short-term pulse-based phenomena, including paired pulse facilitation, post-tetanic potentiation, and spike rate-dependent plasticity, were observed with self-relaxation characteristics. Based on the voltage excitation period, the time scale of the volatile memory can be tuned. Temperature measurement reveals the ion displacement-induced conductance channels become frozen below 220 K. In addition, the volatile analog devices can be configured into nonvolatile memory units with multibit storage capabilities after an electroforming process. Therefore, on the same platform, we can configure volatile units as nonlinear dynamic reservoirs for performing neuromorphic training and the nonvolatile units as the weight storage layer. We proceed to use voice recognition as an example with the tunable time constant relationship and obtain 94.4% accuracy with a minimal training data set. Thus, this iontronic platform can effectively process and update temporal information for reservoir and neuromorphic computing paradigms.

## Introduction

As a result of the significant advancements in artificial intelligence, the internet of things, and big data technologies, a variety of intricate challenges including image and voice recognition, autonomous driving, and natural language processing, have been addressed through the utilization of the prevailing von Neumann architecture.^1−4^ However, this architecture is plagued by higher energy consumption and latency, as the memory and processor are physically segregated. To address this issue, researchers have proposed the adoption of neuromorphic computing (NC) architectures inspired by the human brain, in which the memory and processing elements are colocated, thereby eliminating the energy cost and latency of data shuffling.^5−8^

Among various artificial neural network (ANN) architectures based on SRAM, DRAM, and memristor, the memristor-based ANN has been extensively studied due to its simple fabrication process and low integration cost,^9−13^ and memristors can have volatile or nonvolatile natures, resembling the short-term and long-term memory of the biological brain. In terms of ANN architectures, feedforward neural networks (FFNN) are particularly useful for static spatial patterns, such as image recognition, while recurrent neural networks (RNN) are more practical for dynamic data, including voice recognition and forecasting. RNN requires less number of training parameters compared to the FFNN network, thus reducing complexity.^14^ However, the training of RNNs suffers from a vanishing gradient problem. To solve this issue, a reservoir computing (RC) method was introduced where training updates only at the output layer, thus bypassing the interior complexity and reducing the number of training parameters. There are two main parts in the RC scheme: a reservoir layer and an output layer. The volatile memristors can act as a reservoir of the RC system, whereas the output layer can benefit from nonvolatile elements to retain the training weights.

Some of the existing volatile memories are digital, meaning they set a certain threshold voltage to trigger the conductance change, and they cannot emulate the analog brain functionalities though still good in selector applications and artificial nociceptors.^15−21^ Recently, some researchers have shown analog-type memristor devices, where the conductance can be gradually tuned, thus emulating biological synapse.^22−24^ Midya et al. have demonstrated an RC system to classify the Modified National Institute of Standards and Technology (MNIST) handwritten digits database with diffusive memristors (Pd/SiO_2_/Ag/Pt) and drift memristors(Pd/Ta_2_O_5_/Ta),^25^ even though the accuracy of the implementation was 83%. Moon et al. performed classification and forecasting of temporal data using W/WO_*x*_/Pd/Au volatile memristor,^6^ and a parallel dynamic RC system was investigated by Zhong et al. using Ti/TaO_*y*_/TiO_*x*_/Pt volatile memristors.^26^ In the above-mentioned works, the output layer was trained separately or using a different set of memristors.^27^ In light of these, a universal memristor that can be configured as either volatile or nonvolatile is of utmost importance to reduce footprints and latency in the memristor-based RC system. This would also allow one to realize low-cost RC systems with customizable layouts on the same platform of memristors.

Inspired by natural neurons where the transport of Na and K ions forms the basic neural impulses, we seek solutions in an alkaline ion-enabled solid-state system that is also CMOS compatible. In this research, we report an iontronic device consisting of TiO_*x*_, which is known as a good cathode and anode material, and lithium phosphorus oxynitrate (LiPON), a good solid-state Li electrolyte, together forming a device capable of controllable ionic motion under applied voltages, and configurable to either volatile or nonvolatile behavior. Volatile memory characteristics can be obtained without needing device forming or compliance currents, thus obviating complex setups. The pulse-dependent study revealed short-term plasticity (STP), where conductance gradually varies with the application of short-duration pulses. The time scale of the volatile memory devices is a function of the voltage excitation pulse widths. With a temperature-dependent conduction study, there was a sharp change in conductance above a threshold temperature of 220 K. In contrast, below the threshold temperature, the ion displacement-mediated conductance gives way to weakly temperature-dependent variable range hopping conduction. After electroforming, i.e., creating conductive filaments, these devices can also be configured for nonvolatile memory applications with good retention and endurance. Thus, both volatile and nonvolatile properties can be obtained on the same memristor-based NC chips, a desirable iontronic platform for creating non-von-Neumann architectures. The tunable timing characteristics of devices were applied to a model task of voice recognition, showing excellent accuracy of about 94.4% with a minimal amount of training data input.

## Results and Discussion

### Device Material and Structure Characterizations

**Figure 1:**
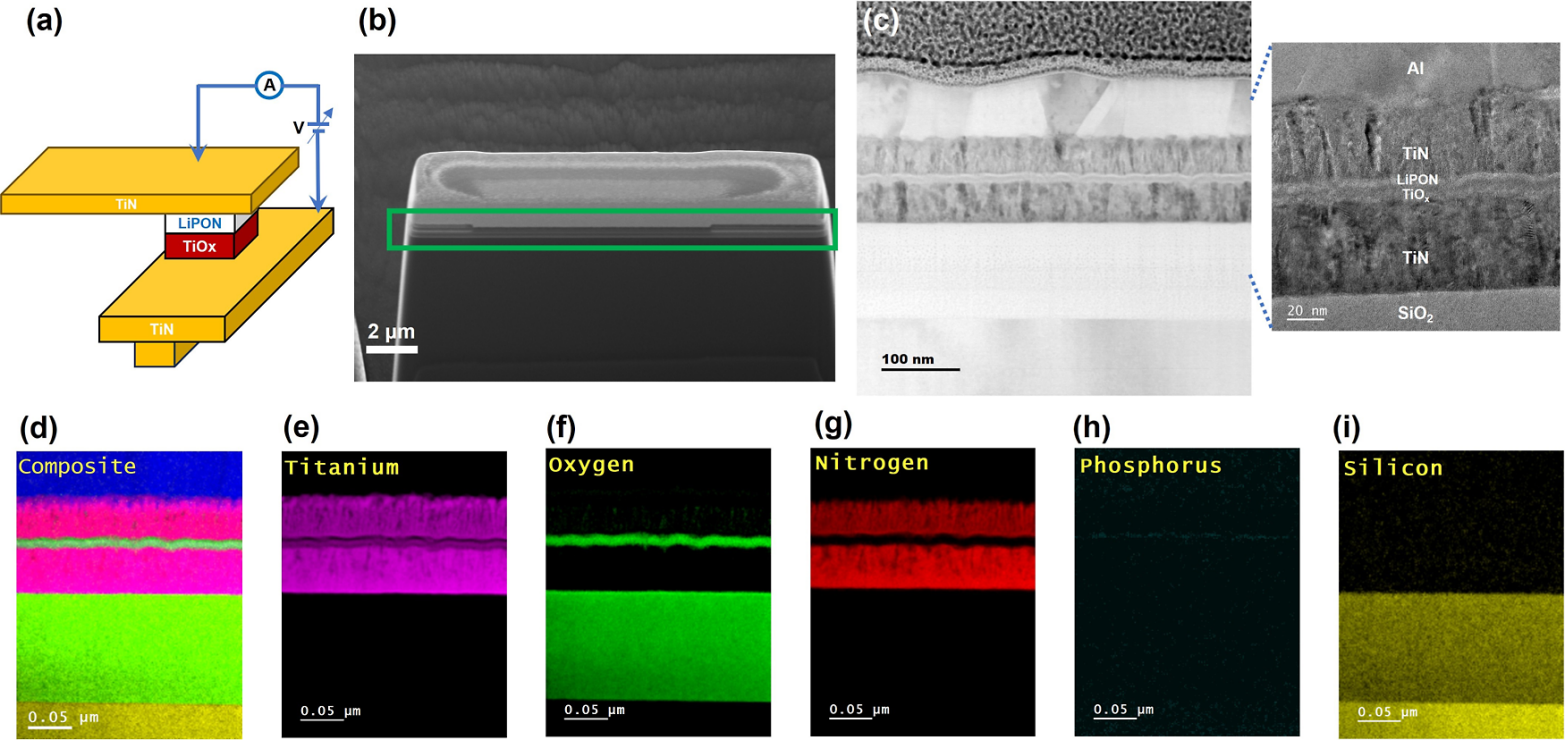
Material characterizations of the memory device. (a) Schematic of the device structure. (b) SEM image of the device lamella profile prepared by FIB showing all layers on the sidewall profile. (c) Cross-sectional STEM micrograph of the device with a zoomed-in TEM image of the device showing several layers present on the device. (d–i) EELS elemental mapping.

A schematic of the device structure, consisting of two TiN layers as top and bottom electrodes, and a TiO_*x*_ and LiPON bilayer as the active switching layer, is shown in Figure 1a. The top electrode is connected to the source whereas the bottom electrode is grounded. Figure 1b shows a lamella with the cross-sectional focused ion beam scanning electron microscopy (FIBSEM) overview of the device layers, which will later be processed for transmission electron microscope (TEM) analysis. The region of interest is shown with a green rectangular box and the active region (thinner part in the middle) is surrounded by side walls of SiO_2_ passivation.

In the TEM images, shown in Figure 1c, the different material layers are contrasted (from bottom to top, SiO_2_ on the substrate, TiN of 50 nm, TiO_*x*_ of 8 nm, LiPON of 4 nm, TiN of 45 nm, Al of 70 nm). Both the top and bottom TiN electrodes are polycrystalline, whereas TiO_*x*_ and LiPON do not show any visible crystallinity, reflecting an amorphous nature. Next, we performed electron energy loss spectroscopy (EELS), shown in Figure 1d–i, and the different elements, including titanium, oxygen, nitrogen, phosphorus, and silicon, are precisely traced in their corresponding layers.

### DC Characteristics

**Figure 2:**
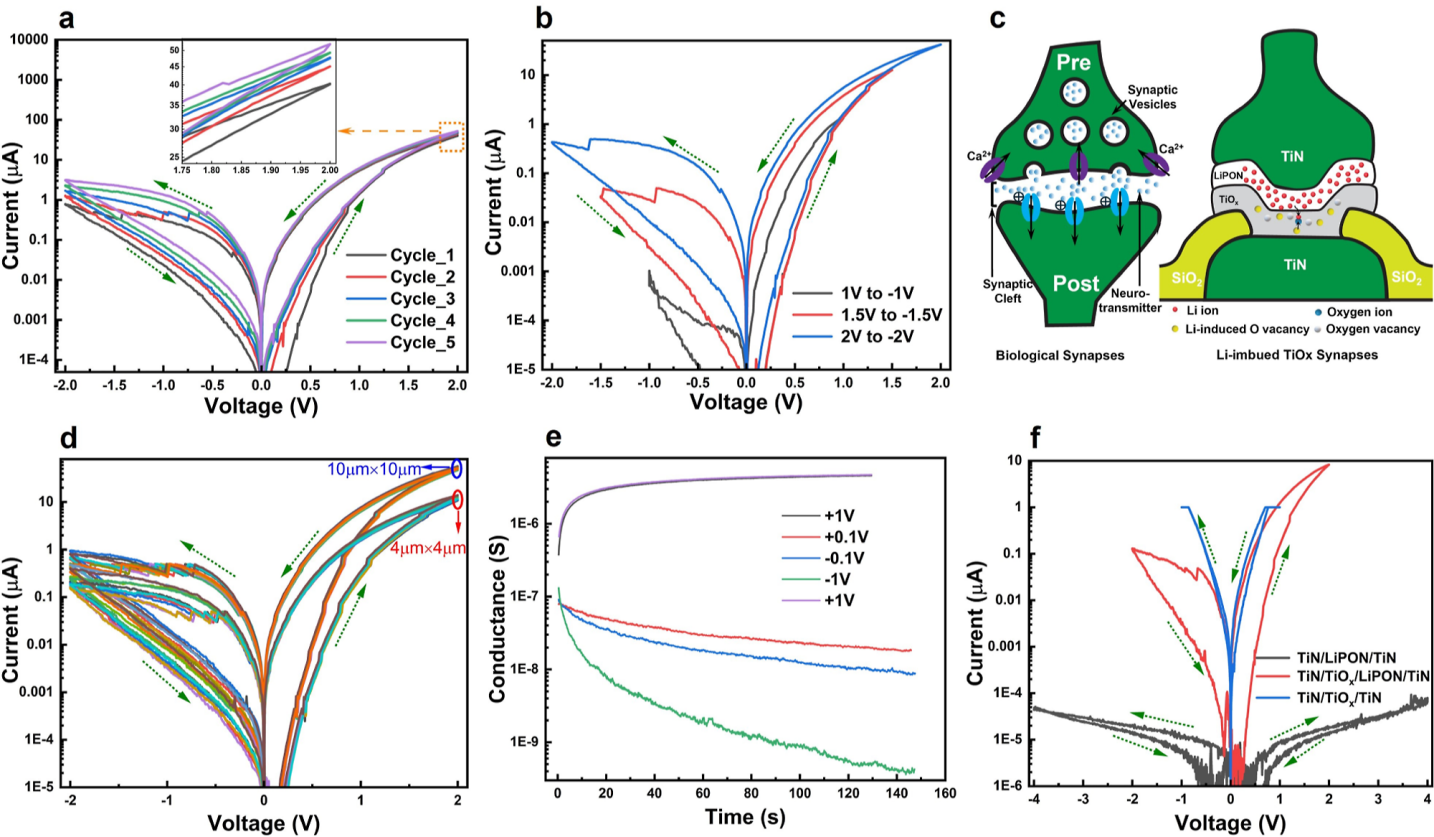
DC characteristics of the Li-infused TiO_*x*_ memory device. (a) A gradual change in *I*–*V* characteristics of the device upon sweeping five full voltage loops, (b) change in conductivity as a function of increasing voltage, (c) analogy between biological synapse and Li-ion imbued TiO_*x*_ synapse, where Li-induces oxygen vacancies are created due to the migration of Li-ion into TiO_*x*_ layer, which then forms a bond with oxygen atom, leaving the TiO_*x*_ more oxygen deficient (reducing the local *x*), (d) *I*–*V* characteristics of ten 4 μm × 4 μm and ten 10 μm × 10 μm devices and conductance scales with the area, (e) self-relaxation and voltage-assisted relaxation from the device (absolute value of the conductance was plotted for negative voltage), (f) *I*–*V* characteristics comparison of different control devices before forming, where the switching layers have the same thickness.

Upon the application of linearly sweeping voltages, the device shows a hysteresis loop, meaning that there is a volatile resistive switching from a high resistance state (HRS) to a low resistance state (LRS) at a positive polarity and from LRS to HRS at opposite polarity, as shown in Figure 2a. The conductance modulation is also a function of sweep speed, as demonstrated in Figure S1a, where a positive current–voltage sweep is plotted with different sweep delays. As the speed decreases, the ratio (i.e., at a read voltage of 0.1 V) of the conductance modulation becomes smaller due to self-relaxation. The reversed rotation in opposite polarities is a clear indication that the volatile conductance change is not from trivial mechanisms such as parasitic capacitance. The inset of Figure 2a shows that repeatedly sweeping full voltage loops increases the overall conductance. The device’s conductance also depends on the amplitude of the applied biases. Figure 2b illustrates the change in the conductance with different amplitudes of voltage biases. This indicates that with a higher applied voltage, more ions can enter and depart the TiO_*x*_ layer and alter the conductance more. Figure S1b shows the *I*–*V* characteristics of five consecutive positive sweeps (0 V (+2 V)–0 V) and five consecutive negative sweeps (0 V–(−2 V)–0 V). When the voltages sweep only in one polarity (half loops), the devices’ conductance increases after consecutive positive sweeps and decreases after consecutive negative voltage sweeps, as shown in Figure S1b. With repeated positive potentials, more Li-ion can migrate into the TiO_*x*_ layer and create defects, causing the increase in conductance; and repeated negative potentials do the opposite by depleting ions. This gradual change in conductance is analogous to the efficacy modulation in biological synapses upon the application of the repeated stimulus. Thus, Li-ion modulated TiO_*x*_, before forming, exhibits gradual and volatile conductance modulation and can be used for artificial synapses.

When an action potential is generated at the presynaptic neuron terminal or axon, voltage-gated Calcium channels trigger and Calcium ions (Ca^2+^) start flowing into the presynaptic neuron and synaptic vesicles shift toward the synaptic cleft and release neurotransmitters, which then bind to receptor proteins across the gap and cause Na or K ions to conduct through the postsynaptic neuron, and this process strengthens or weakens the connection strength between synapses. Similarly, in Li-imbued TiO_*x*_ iontronic devices, under positive applied bias, lithium ions (red spheres) can migrate into the TiO_*x*_ layer as shown in Figure 2c. Here, the yellow spheres represent Li-ion-induced vacancies, and the gray ones are native oxygen vacancies already in TiO_*x*_. Li ions preferably combine with oxygen and chemically reduce Ti, which has similar effects as forming oxygen defects in TiO_*x*_ (reducing *x*). Therefore, the Li-ion insertion/extraction increases/decreases the device conductivity accordingly. Li migration is similar to oxygen migration but it is much easier and happens readily at room temperature and low current densities, i.e., no set/reset voltages/currents or current induces hotspots are needed. The current amplitude can vary well over an order of magnitude in these processes, depending on the history. The devices therefore possess history-dependent volatile memory suitable for neuromorphic applications.

The conduction of the device scales well with the device area, which indicates a nonfilamentary type of conduction. Figure 2d shows a total of 20 devices’ IV characteristics with two different device sizes: 10 μm × 10 and 4 μm × 4 μm. Also, Figure S1c shows 50 more (6 μm × 6 μm) devices’ IV characteristics. As can be seen from those figures, there is little device-to-device variation as no forming or filament is needed, depicting the good uniformity of the device’s volatile characteristics. Moreover, the conductance is proportionally larger for larger area devices. Figure S1d represents the *I*–*V* characteristics of the volatile devices for 100 cycles. The devices can sustain repeated cycling without much degradation, representing the robustness of the devices.

Another important phenomenon observed from the devices is their self-relaxation behavior. To understand this, several systematic tests were performed. First, we applied a very small read voltage of −0.1 V to check that there was no conductance change from the fresh device. Once we applied +1 V to the device, the conductance increased (changed from HRS to LRS), indicated by the black color curve in Figure 2e. Next, a small read voltage of −0.1 V (blue color) and +0.1 V (red color) were applied to capture the self-relaxation toward lower conductance, confirming the volatility of the memory device. A higher negative bias of −1.0 V shows similar but voltage-assisted relaxation to a much lower conductance state (green color curve), which largely accelerates the relaxation process and can be a way to speed up the system for practical applications. After changing the polarity back, the conductance returns to the original behavior, represented by the violet color curve. It is worth noting that due to the cathodic nature of TiO_*x*_, the center voltage of the ion insertion/extraction is not at 0 V, which leads to the monotonic conductance drift between cycles depending on the voltage sweep window. As an example, when the negative voltage is not enough to fully extract the ions, the corresponding conductance drifts up appreciably from cycle to cycle (Figure 2a). When sweeping asymmetrically instead (−2.7 to 2 V, Figure S1d), the cycle-to-cycle drift becomes negligible. Therefore, the thermodynamic center of the system is approximately at −0.35 V, indicating a spontaneous redistribution of Li ions due to chemical potentials. This is also the origin of the system exhibiting faster conductance change at 1 V than at −1 V (Figure 2e).

The strong volatile behavior in these devices is a combined result of the electrolyte LiPON and the cathodic TiO_*x*_. To verify this, we fabricated control devices with only TiO_*x*_ and only LiPON of the same 12 nm thickness, and the characteristics are shown in Figure 2f. The LiPON-only devices are extremely resistive and exhibit different hysteresis changes (dashed arrows, curve rotation is different from the volatile changes) due to lagged charging and discharging of their capacitance with large time constant. Whereas the TiO_*x*_ only devices before forming have small hysteretic behavior because the TiO_*x*_ is populated with some oxygen vacancies that can also migrate especially under electric fields, and more so in hot spots (such as during the forming process). The LiPON/TiO_*x*_ hybrid devices have intermediate conductance as expected, and the much stronger volatile change can be attributed to their mutual influence. When LiPON was grown with reactive sputtering from the Lithium phosphate target, some Li ions were already incorporated inside the TiO_*x*_ layer and created ionic defects ready for voltage manipulation. LiPON functions as a Li source and reservoir and can absorb or release ions at applied biases. TiO_*x*_ functions as the cathode and its conductivity varies with the concentration of oxygen vacancies.^28,29^ Also, TiO_*x*_ is often used as electrodes in rechargeable batteries where Li ions can travel in and out of the nanostructures, easily forming intercalated ion storage and reducing TiO_*x*_.^30−33^ With the ion intercalation and modified TiO_*x*_ stoichiometry, the conductivity of our devices increases or decreases accordingly, analogous to the synapse systems in its stimulated conductivity modulations.

### Pulse Characteristics

To emulate the brain-like synaptic functions, pulse-dependent characteristics from the devices are essential as most of the signals in the biological brain are similar to short pulses. Synaptic plasticity, which is defined as the proficiency of neurons to adjust the level of connection strength between them, is one of the most important aspects of NC.^34^ In biological synapses, once an action potential is generated at the axon, it induces a Calcium ion influx into the presynaptic neuron and releases neurotransmitters. These neurotransmitters then migrate to the postsynaptic receptors temporarily modifying the synaptic efficacy. If there is another identical action potential generated before the recovery time of the calcium ions, the response from the postsynaptic neuron becomes higher compared to its first one. This effect is known as paired-pulse facilitation (PPF).^35−37^ Likewise, a train of consecutive identical pulses of a certain duration result in a gradual augmentation of synaptic plasticity, which is known as post-tetanic potentiation (PTP).^38^

**Figure 3:**
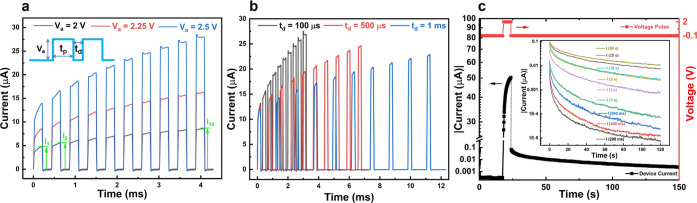
Device behavior upon the application of pulse voltage. (a) The device’s response upon varying pulse amplitude and inset depicts the key parameters: *V*_a_, pulse amplitude; *t*_p_, pulse duration; *t*_d_, pulse delay. (b) Device current upon varying pulse delay. (c) Continuous bias and its corresponding conductance. Inset shows the conductance decay at different excitation durations.

The inset of Figure 3a shows the key parameters of the applied pulses on the devices, including pulse amplitude *V*_a_, pulse duration *t*_p_, and pulse delay *t*_d_. The indexing of the PPF is defined as the ratio of the current value of the second pulse relative to the current value of the first pulse, whereas that of PTP is represented by the ratio of the current value of the 10th pulse to the current value of the first pulse. We first vary the pulse amplitudes to observe their effects on device conduction. As can be seen from Figure 3a, when the applied potentials are higher in amplitude, both the PPF and PTP increase much faster. This fact can be explained by the amount of ions participating inside the device is higher when the electric field is higher, resulting in a much higher conductance increase. The potentiation and depression characteristics are shown in Figure S2a, where the pulse amplitude, pulse width, and pulse interval are kept the same. The first 50 pulses are positive and result in potentiation (conductance increase) from the device and the next 50 pulses are negative and result in depression (conductance decrease) characteristics.

Synaptic plasticity is a function of the spike rate as well. Spike rate modifies the number of neurotransmitters from the presynaptic neuron, which modifies the strength of synapses; this process is known as spike-rate dependent plasticity (SRDP). Analogous to biological synapses, the conduction also depends on the delay between pulses as the competing self-relaxation behavior exists. To test this assumption, consecutive voltage pulses with a certain delay are applied to the device and corresponding currents are measured and plotted in Figure S2b. The applied pulse voltage is 2 V and the pulse width is 0.5 ms, whereas the pulse interval is 15 ms. With the high pulse interval value, some ions relax back and the conductance increase is slower. The aero in Figure S2b indicates the change in the conductance values. This is why a longer delay results in a smaller final current despite the accumulated pulse time remaining the same, which is depicted in Figure 3b. Thus, Li-ion imbued TiO_*x*_ shows the SRDP of a biological brain and can be used for artificial synaptic devices.

A continuous biasing approach was performed to determine the time scale of the volatile memory device. To capture the decay after excitation, we incorporated continuous bias of different time intervals and amplitudes (for example, 2 V for 5 s was used to capture the potentiation; and to capture the decay, a small negative voltage, −0.1 V, was used). Figure 3c shows the overall voltage pulse and corresponding current. Also, the inset of Figure 3c shows that a larger duration of voltage stress results in slower conductance decay, which can be attributed to a large number of Lithium-ion migrations. Next, the current decay (durations of 1 s to capture only the relevant, fast relaxation) was fitted with an exponential decay and extracted the time scales. The extracted decay constant is then plotted as a function of the excitation pulse widths. As can be seen from Figure S2c, the fitted results can be approximated with a power-law relationship, meaning the short-term memory can be tuned with the excitation duration, which was used for voice recognition applications as described in the following sections. Thus, our device shows *tunable volatile memory* features.

### Temperature Dependent Characteristics

**Figure 4:**
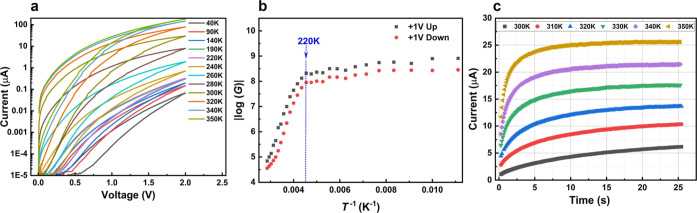
(a) Temperature-dependent current–voltage characteristics (b) resistance value at +1 V for two different sweep conditions: one at up sweep and another is down sweep. (c) Constant-voltage stress at different temperatures with an applied voltage of +1 V.

The participation of ions can be distinctly identified by varying the device temperature as ions tend to freeze out easily at reduced temperatures. The temperature was varied over a wide range, from 40 to 350 K, while the voltage sweep was fixed from 0 to 2 V (up sweep) and then 2 to 0 V (down sweep). Figure 4a shows that before the temperature reached a certain threshold value, the IV loop openings were small and device behavior did not change much, but changes were more pronounced above the threshold temperature, meaning some thermally activated conduction channels are now participating. To determine the threshold value, we extract the discrete current data value at +1 V at both up sweep and down sweep and plot it as a function of the inverse of temperature, i.e. 1/*T*. There is a clear slope change at 220 K as shown in Figure 4b. This transition can be attributed to lithium ions becoming frozen below this threshold temperature, which is close to the reported freezing temperature of Li ions. Therefore, only thermally promoted Li ions can migrate. On the other hand, the relative change under sweeping voltage is a result of the applied electric fields driving the already promoted ions, therefore not as temperature dependent. Similarly, Figure S3a shows that the absolute relative change of current value between up sweep (*I*_up_) and down sweep (*I*_down_) at +1 V is increasing above the transition temperature.

The slope of the semilog plot of the conductance with respect to 1/*kT*, which is known as the activation energy barrier, was calculated from Figure S3b, where the conductance values are recorded above the transition temperature. The activation barrier was found to be around 0.20 eV, which approximately corresponds to the density functional theory (DFT) calculated value (a low energy barrier of around 0.4 eV). In the O-defective TiO_2_ supercell shown in Figure S3c, the green spheres along the path represent lithium (Li) atoms at different positions, illustrating their optimized migration path, i.e., the most energetically favorable path for Li-ions. The graph in Figure S3d shows the energy barrier for Li migration along the reaction pathway shown in Figure S3c. The energy barrier peak at around 0.4 eV indicates the maximum energy required for Li to migrate between two local minima. This low energy barrier suggests that Li ions can move relatively easily in the O-defective TiO_2_ structure, implying high ionic conductivity, which is crucial for low-operating voltage devices. This energy barrier is comparable with the reported energy barrier for pristine anatase TiO_2_ structures.^39^ Now, below the transition temperature, the Li ions are frozen as there is a minor conductance change. Figure S3e illustrates the absolute log value of conductance as a function of the temperature (*T*^–1/4^). A linear relationship between conductance and the temperature (*T*^–1/4^) can be seen from Figure S3e, indicating that the conduction mechanism is mostly dominated by variable-range hopping (VRH), which is much less temperature-dependent. This conduction mechanism is similar to reported Mott VRH at low temperatures.^40,41^

The constant voltage stress curves above the threshold temperature are shown in Figure 4c. The applied voltage was +1 V. Higher temperature leads to higher final conductance and faster conductance change. Faster ionic motion is expected for higher temperatures, therefore the system can reach dynamic equilibrium significantly faster. The temperature is a useful controlling knob for the system if tuning operation speed is necessary.

### Nonvolatile Characteristics

**Figure 5:**
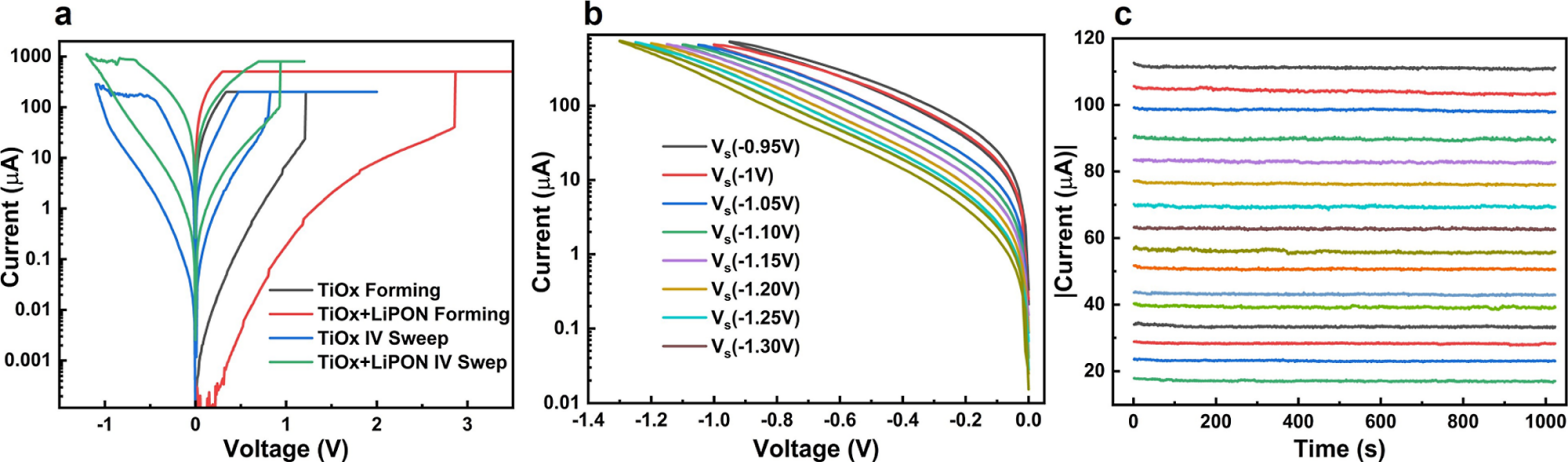
(a) Forming process comparison of TiO_*x*_ and Li-imbued TiO_*x*_ memory devices, (b) multistate capability of the proposed device when stopped at different RESET stop voltages, which is denoted by *V*_s_, (c) retention behavior of the 16 states from the memory device.

The same volatile memory device can be configured into a nonvolatile one by an electroforming process. The forming process introduces conductive filaments in Li-imbued TiO_*x*_ resistive switching devices, and the device is SET to LRS. The device can be RESET to HRS by applying opposite polarity voltages. We compare the electroforming behavior of hybrid 8 nm TiO_*x*_/4 nm LiPON devices with pure 8 nm TiO_*x*_ control devices, shown in Figure 5a, and the former need higher forming voltage and larger compliance current to form appropriate conductive filament width. This is reasonable as LiPON is a good electrical insulator and a large fraction of the overall applied voltage is dropped across this layer instead of TiO_*x*_.

After forming the devices, we perform the RESET and SET operations for both devices. For the reset operation, the voltage was kept at a fixed value rather than a compliance current as current may vary from device to device. As clearly shown in Figure 5a, the Li-infused TiO_*x*_ becomes nonvolatile after filament formation. Overall, these devices have higher conductance despite the total layer thickness being higher, because TiO_*x*_ becomes more defective in the presence of Li ions, and LiPON likely experiences dielectric breakdown and no longer participates.

Interestingly, this nonvolatile device can be reset to multiple resistance states without overlapping in between, meaning it is capable of storing multibit information. As we stop at different reset voltages, the current from the device is gradually reduced, as shown in Figure 5b. Figure S4a represents the retention characteristics after each reset test of the device. As can be seen from the figure, the devices retain more than 32 states for thousands of seconds without much noticeable change. This ensures sufficient margins for programming 4-bit (16-state) information on each device as shown in Figure 5c, which will be assumed in the next session for the proposed circuit integration.

Figure S4b depicts 20 nonvolatile Li-imbued TiO_*x*_ devices’ IV characteristics. There is some device-to-device variation, which is reasonable for TiO_*x*_-based devices fabricated with in-house facilities. It is worth pointing out that the Li-containing devices’ transition from volatile to nonvolatile is irreversible, and once the conductive filament is formed, the system behaves very similarly to pure TiO_*x*_ devices with nonvolatile memristor behavior.

### Memristor-based Reservoir Computing

**Figure 6:**
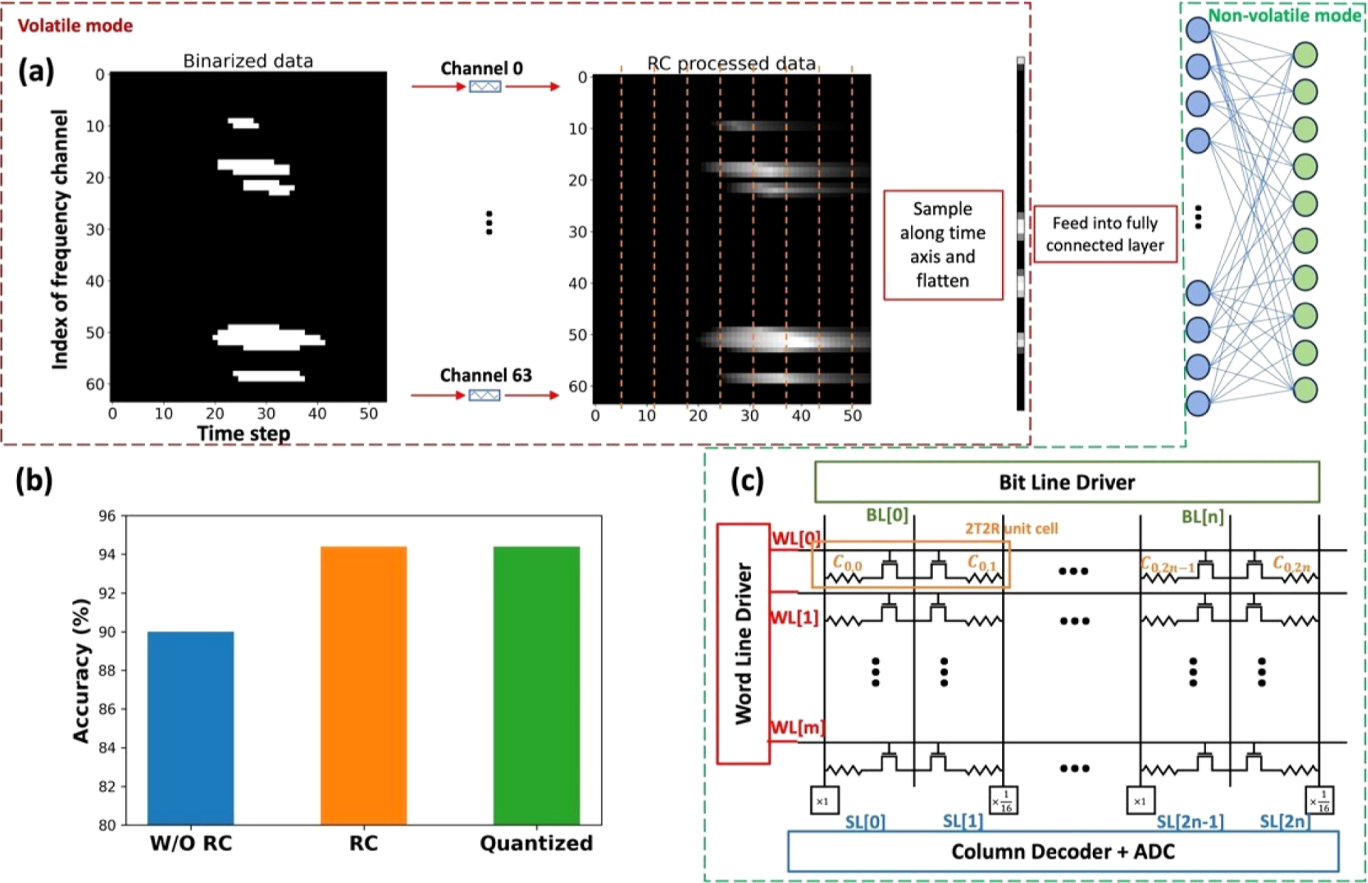
Voice recognition task using proposed memory. (a) Process flow of RC for voice recognition. (b) Comparison of classification accuracy between quantized RC model, RC model and model without RC processing. (c) Schematic of proposed 2T2R CIM array.

RC is a computation framework that maps input signals to higher dimensional space, through a nonlinear mapping “reservoir”. “Reservoir” can be a dynamic system that evolves in time, which can be expressed as *f*(*t* + 1) = *f*(*t*, input). The volatile switching behavior of our fabricated device, however, shares similar characteristics with a dynamic reservoir: the future resistance state, *f*(*t* + 1), is dependent on the applied voltage (input) and current resistance state, *f*(*t*). To illustrate the neuromorphic capabilities of our devices, a physical RC system is proposed for voice recognition tasks. The workflow of the RC system is illustrated in Figure 6a. After preprocessing the raw voice data using Lyon’s passive ear model, the generated Cochleagram (frequency channel vs time) is normalized and binarized (with a threshold value of 0.2). The Cochleogram of original voice data and binarized one with a threshold value is shown in Figure S5a. The data vector of each frequency channel (along the time step axis) is converted into a voltage input vector (with data “0” representing 0 V and data “1” representing 2 V), and the time step of the voltage input vector is 33 ms. The voltage input vector of each channel is fed into the volatile device connected with the channel. The Cochleogram of the RC processed data from binarized input data is shown in Figure S5a, which almost perfectly resembles the original voice data. Figure S5b represents the voltage input of a frequency channel and the corresponding current from the volatile memory device. As can be seen from the figure, the conductance of the volatile device will change (potentiate or self-relax) based on the voltage input vector. Next, the conductance value of each device is evenly sampled along the time axis (8 data points sampled in our simulation). Sampled conductance values of 64 channels are flattened into a 1D feature vector and fed into the fully connected layer to generate the classification results.

The whole system has been trained for 300 epochs, the classification accuracy of the RC model, defined as the average accuracy of the 10-fold cross-validation, is 94.4%, which outperforms the binary data without RC processing using the same network architecture by 4%, as shown in Figure 6b. If we also use the conventional constant time scale assumption, the simulated accuracy can well exceed 97% for this particular task. The accuracy improvement is achieved without computation overhead. A performance comparison between existing literature and our demonstrated device is shown in Table S1. It is clear from the table that our devices show tunable volatility and multibit nonvolatile capability, which is special in a single device. Our platform excels with great device tunability and platform customizability.

The fully connected layer discussed above is based on software simulation, each weight inside the layer is stored in the format of a 32-bit floating point number (float32). Arithmetic operations as well as storage of floating-point numbers are resource-intensive and not energy efficient for hardware implementation. To explore the possibilities of scaling down the representing bits of weights, post-training quantization (PTQ) is used to map the trained weights from float32 to int8. The quantized model is validated using the same validation data set, and the results show that there is no accuracy drop after quantization. Based on that, a 2T2R compute-in-memory (CIM) array is proposed (shown in Figure 6c) to accelerate the operation of vector-matrix multiplication, which is used inside the fully connected layer, with our devices working in nonvolatile mode.

With each device programmed to store 4-bit data, the 2T2R unit cell consists of two nonvolatile devices and two access transistors. Two nonvolatile devices inside the 2T2R unit cell are used to store one 8-bit weight, the left device is used to represent the most significant 4 bits (MSB) and the right is used to represent the least significant 4 bits (LSB). Both devices share the same bit line signal (*V*_BL_*n*__), therefore, current flowing through the MSB source line (*I*_SL_2*n*–1__) would be
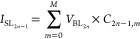
while the current for LSB line (*I*_SL_2*n*__) is
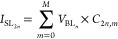
*I*_LSB_ is fed through a 1/16 current mirror so that the sum of *I*_MSB_ and *I*_LSB_ can be used to represent an 8-bit value. The current sum, (*I*_MSB_ + 1/16 * *I*_LSB_), is sampled by ADC, which would be digitalized and output the 8-bit product results.

## Conclusions

In summary, we have demonstrated a Li-ion imbued TiO_*x*_ iontronic device, with both volatile and nonvolatile memory capabilities. Before forming, the devices show inherently uniform gradual conductance change upon the application of external bias due to the controlled ionic motion. A self-relaxation phenomenon confirms the volatile nature of the memory. The Li-ion-induced vacancy creation inside TiO_*x*_ resembles Calcium-ion-induced synaptic modulation inside biological synapses. Pulse-dependent results strongly resemble the different brain functionalities such as PPF, PTP, and SRDP characteristics. The time scale of the volatile memory devices can be tuned by changing the voltage pulse widths. In addition, the same volatile device can be transformed into nonvolatile memory by an electro-forming process. To illustrate their performance, we applied both tunable volatile and nonvolatile versions of the devices to a proposed RC architecture and performed voice recognition tasks with over 94% accuracy. Therefore, we’ve demonstrated a general iontronic platform that is customarily configurable to mimic brain functions on a hardware level.

## Experimental Methods

### Device Fabrication and Characterization Steps

Three-inch Si wafers with thermally grown 100 nm SiO_2_ were used as the substrates. After finishing the cleaning process, the TiN bottom electrode was deposited using DC sputtering. It is then patterned with standard photolithography processes and etched using ion milling with a secondary ion mass spectroscopy to detect the end point. Next, a 60 nm SiO_2_ passivation layer was deposited using the plasma-enhanced chemical vapor deposition process. Then the sample was again patterned using photolithography and the SiO_2_ layer was etched using reactive ion etching to open active areas of the devices. Another lithography and liftoff step defined the switching layer and the top electrode (TiN/Al) and formed junctions with the bottom electrode through the active openings. The LiPON and TiO_*x*_ were reactively sputtered by properly adding N_2_ and O_2_ gases, respectively. FIBSEM and TEM were done using ZEISS SEMFIB and JEOL TEM.

### Electrical and Temperature Measurement Steps

All electrical measurements, including DC and Pulse, were done with a Keithley 4200A semiconductor parameter analyzer, equipped with source measure units for DC measurement and pulse-measurement units and remote preamplifier/switch modules (RPM) for pulse measurements. Temperature-dependent measurement was performed using a Janis probe station.

### DFT Calculations

The DFT study was conducted using the Vienna Ab-initio Simulation Package (VASP) to optimize the structure of anatase TiO_2_ to its ground state, with the ultimate goal of determining the energy barrier for Li migration.^42^ Initially, the structure of anatase TiO_2_ underwent relaxation with a kinetic energy cutoff of 450 eV for the plane wave. Brillouin zone integration was conducted using the Monkhorst–Pack scheme, employing a *k*-point mesh of 5 × 5 × 5, with the exchange–correlation functional approximated using Perdew–Burke–Ernzerhof (PBE) within the generalized gradient approximation.^43^ Under the same conditions, a four times larger supercell was used to find the local minima for the Li-doped TiO_2_ O-defective before the climbing image nudge elastic band (CI-NEB) calculation.^44^ The CI-NEB calculation was carried out using high-performance computers equipped with GPUs.^45,46^ To strike a practical balance between convergence and accuracy, the migration path consisted of five images.

### Voice Recognition Configuration

The voice data was converted into a cochleagram (frequency channel vs time) using Lyon’s passive ear model. After conversion, the processed data consists of 64 channels, each of which would be the input vector applied to the reservoir. To reduce the training data budget, input vectors are binarized first with a threshold value. Each data point of the binarized input vector would be the voltage applied to the physical reservoir, with “1” representing 2 V and “0” representing 0 V (Figure S5b). After RC processing, we again shrink the data size by evenly sampling eight data points on the time scale and fed into a fully connected layer for classification. The fully connected layer is pretrained and optimized using the RMSprop method, and softmax is used to convert the output of the fully connected layer to the classification result. 10-fold cross-validation is used to train (450 samples) and validate (50 samples) the RC system.

## Data Availability

The data that support the findings of this study are available from the corresponding author upon reasonable request.

## Supporting Information Available

Relationship between conductance change and sweep delay, conductance change upon consecutive positive or negative sweep, current–voltage characteristics of 50 volatile devices, endurance test of the device, potentiation and depression characteristics of the device, pulse delay and conductance decay, relationship between conductance decay and input voltage pulse widths, relative values of the current changes at different temperatures, slope above transition temperature, Li migration pathway in the O-defective structure of anatase TiO_2_, energy barrier profile for Li migration, slope below transition temperature, retention characteristics of the nonvolatile device, 20 nonvolatile Li-imbued TiO_*x*_ devices’ IV characteristics, cochleagram of voice data, binarized data, and RC processed data, voltage input of a channel and corresponding current of volatile device at time scale, and performance parameter comparison with other works (PDF)
