# Supplementary material for: Volatile and Nonvolatile Programmable Iontronic Memristor with Lithium Imbued TiOx for Neuromorphic Computing Applications
Source: ACS Nano. 2024 Aug 7;18(33):22045–54. doi: 10.1021/acsnano.4c05137 (PMC11342358; doi:10.1021/acsnano.4c05137)
Supplement: Supplementary file 1 — nn4c05137_si_001.pdf [file nn4c05137_si_001.pdf]

Supporting Information

Volatile and Nonvolatile Programmable Iontronic Memristor with Lithium Imbued TiO_x for Neuromorphic Computing Applications

Rabiul Islam,[1,2,] Yu Shi,[1,2] Gabriel Vinicius de Oliveira Silva,[1,2] Manoj Sachdev,[1] and Guo-Xing Miao[1,2,]*

[1] Department of Electrical and Computer Engineering, University of Waterloo, Waterloo, Ontario, N2L 3G1, Canada

[2] Institute for Quantum Computing, University of Waterloo, Waterloo, Ontario, N2L 3G1, Canada

*Corresponding author email: guo-xing.miao@uwaterloo.ca

1. Current Voltage Characteristics of the Li-imbued TiO_x Devices

Figure S1 represents the DC current voltage characteristics (IV) of the Li-imbued TiO_x devices. The conductance modulation of the device is a function of the sweep delay. **Figure S1a** shows the IV characteristics with different sweep delay, ranging from 10 ms to 0.5 seconds delay between voltage sweeps. As the delay increases, the hysteresis of the memory device (i.e. at read voltage of 0.2 V) decreases, indicating more relaxation towards quasi-static states. **Figure S1b** also illustrates five consecutive IV characteristics of the devices in positive sweep directions (*i. e.*, 0 V – (+2 V) – 0 V) and negative sweep directions (*i. e.*, 0 V – (-2 V) – 0 V). As can be seen from figure that when the device was continuously cycled in the positive sweep directions, it shows monotonous increase in conductivity, whereas consecutive negative sweeping reduces the conductivity. **Figure S1c** shows IV characteristics of fifty volatile devices, which are distributed across 1cm-by-1cm area. This indicates the excellent uniformity of the devices. The devices were SET followed by RESET for 100 times, and the resultant IV characteristics is shown in **Figure S1d**. There is negligible conductance drift under a suitable asymmetric voltage window, representing great endurance.

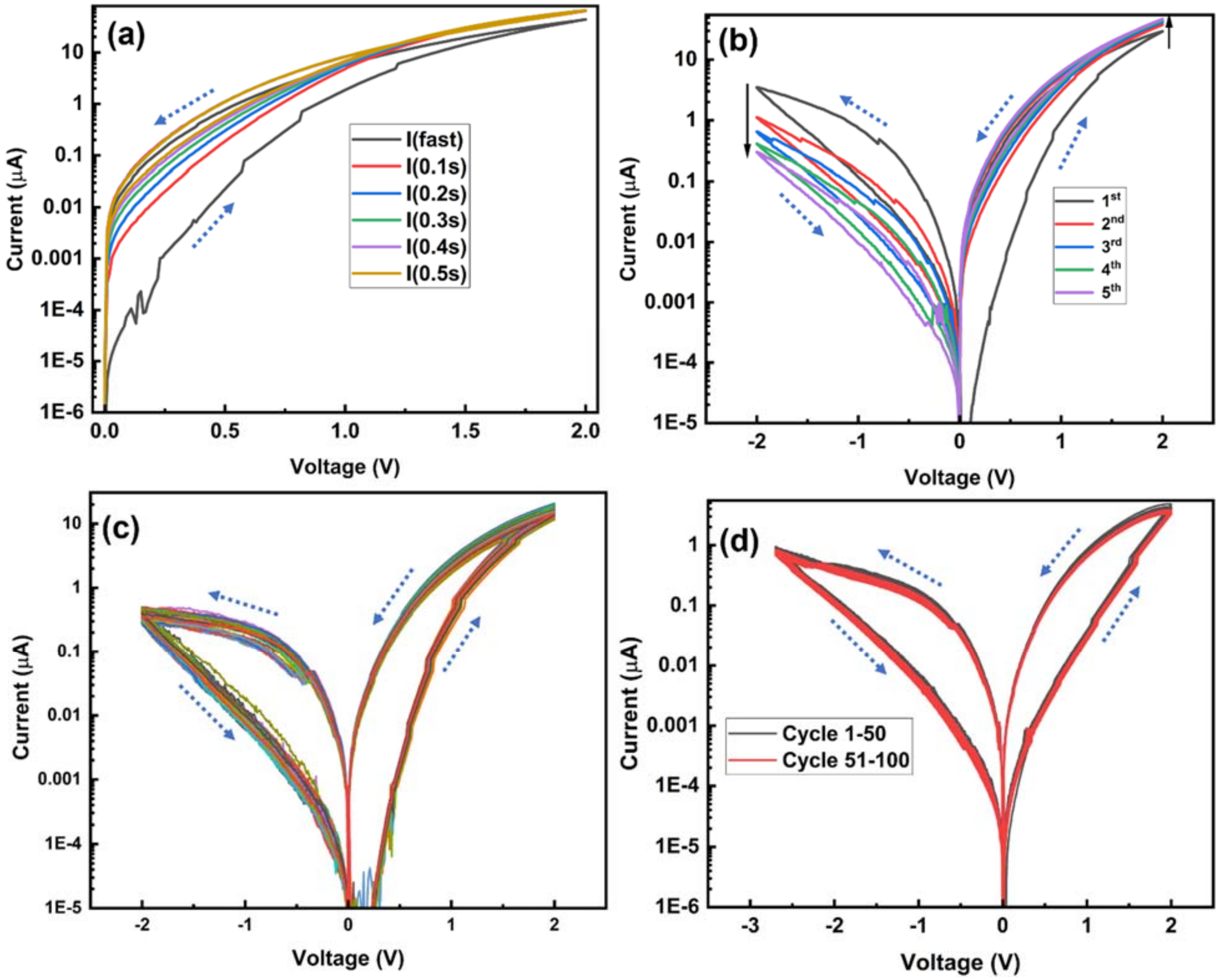

Figure S1. (a) Conductance changes as a function of sweep delay between applied voltage. (b) Gradual increase in conductance upon the applications of five consecutive positive sweep (0 V - (+2 V) – 0 V) and decrease in conductance upon the applications of five consecutive negative sweep (0 V - (-2V) – 0 V). (c) Current-voltage characteristics of 50 volatile devices distributed across 1cm-by-1cm area. (d) Endurance test of the device with asymmetric voltage sweep.

2. Pulse Characteristics of the Li-imbued TiO_x Devices

Figure S2 summarizes the voltage pulse dependent conductance change and the variation of decay time scale with excitation pulse widths. **Figure S2a** represents the potentiation (setCurrent) and depression (resetCurrent) characteristics of the device. The input voltage for potentiation is 2.5 V with a pulse width of 0.5 ms and for depression is -3.5 V with a pulse width of 0.5 ms. Like DC Sweep, consecutive positive pulse excitation (SET pulses) results in augmentation in conductance, known as potentiation, and consecutive negative pulse excitation (RESET pulses) results in decrease in conductance, known as depression. **Figure S2b** shows the input voltage pulses, the delay between pulses, and corresponding current from the device. The applied pulse voltage is 2 V with pulse width of 0.5 ms, whereas the pulse interval is 15 ms. As can be seen from the figure, during the 15 ms pulse interval, the conductance decreases between pulses due to self-relaxation of ions. The arrow indicates the changes in the conductance values. Conductance decay is also a function of excitation pulse widths. After applying a 2 V excitation voltage for a certain duration, the conductance decay was continuously monitored at -0.1 V and fitted with a simple exponential function to extract the initial decay time constant. These decay time constants are plotted with respect to the corresponding excitation duration and shown in **Figure S2c**. The curve best fitted to a general behaviour of $0.45*(t)^{0.25}$. This excitation-dependent-relaxation is used in the later neuromorphic simulations to better represent the system's true behaviour.

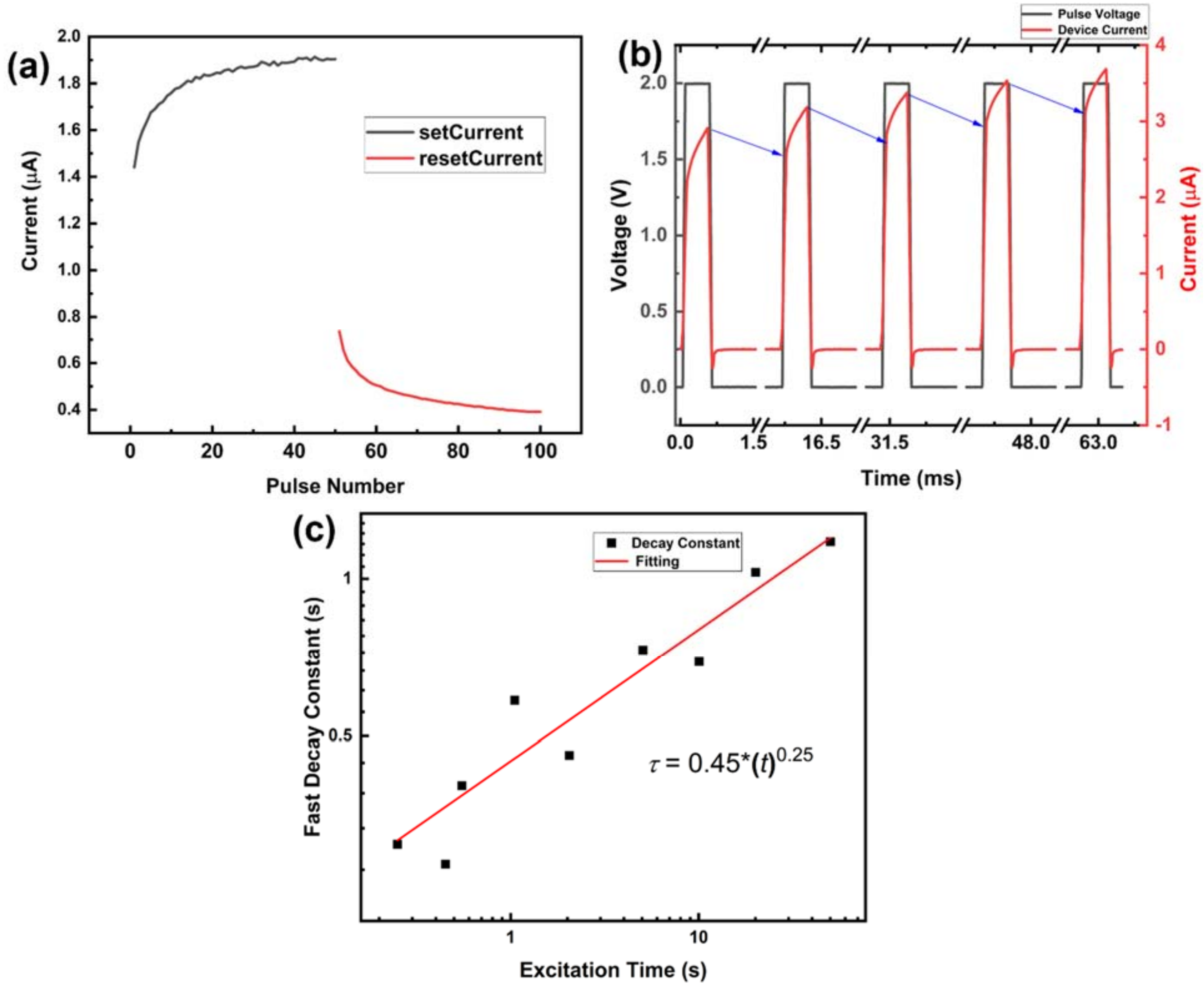

Figure S2. (a) Potentiation and Depression characteristics from the device upon the application of positive and negative voltage pulses. (b) Partial conductance decay when the pulse delay is of 15ms. (c) Power-law fitting result of the conductance decay as a function of excitation duration.

3. Temperature Dependent Conductance Characteristics

Figure S3 represents the extracted current values at +1 V for different temperatures and both for the up (0 V - 2 V) and down sweeps (2 V - 0 V). The relative difference between sweeps is plotted in **Figure S3a**, representing the volatile hysteresis. Above the transition temperature (220 K), the volatile hysteresis is more pronounced. **Figure S3b** represents the conductance values of both up sweep and down sweep above the transition temperature. Upon fitting both data values, the activation energy barrier of 0.21 eV and 0.20 eV was found for up sweep and down sweep, respectively. In the O-defective TiO_2 supercell shown in **Figure S3c**, the green spheres represent the optimized migration path of lithium (Li) ions, *i.e.*, the most energetically favorable path. The graph in **Figure S3d** shows the energy barrier for Li migration along the optimum pathway shown in **Figure S3c**. The energy barrier is around 0.4 eV for Li to migrate between two local minima. **Figure S3e** shows the absolute log value of conductance below the transition temperature as a function of the temperature ($T^{-1/4}$). The $T^{-1/4}$ dependence indicates the conduction mechanism is mostly dominated by variable-range hopping (VRH) when ion motions are frozen.

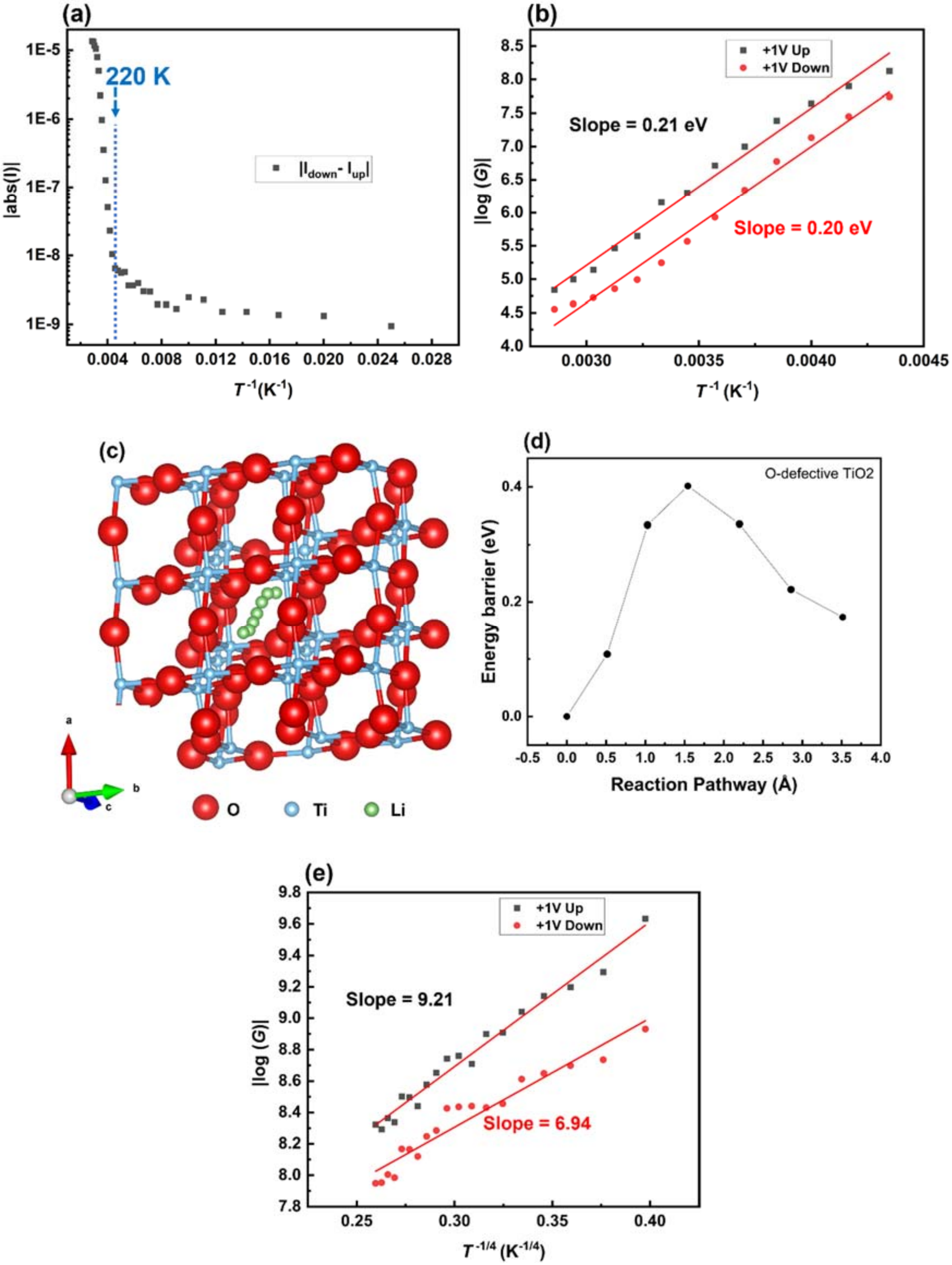

Figure S3. (a) Relative values of the current changes at different temperatures @+1 V. (b) Slope above transition temperature. (c) Li migration pathway in the O-defective structure of anatase TiO_2 using ionic radii style. (d) Energy barrier profile for Li migration

obtained from DFT-NEB (Density Function Theory – Nudged Elastic Band) calculations, showing a barrier of 0.4 eV. (e) Slope below transition temperature.

4. Nonvolatile Memory Characteristics

Figure S4 summarizes the retention behaviour and device-to-device variation. Followed by progressive RESET sweep (for example: 0 V – (-0.66 V) – 0 V), the retention behavior was monitored by applying a constant bias of -0.2 V for 1000 seconds (**Figure S4a**). **Figure S4b** illustrates twenty devices' IV characteristics, where the SET operations of the devices were done using positive sweep and the RESET operations of the devices were done using negative sweep. As can be seen from the figure that the non-volatile version of devices has more device-to-device variation due to their stochastic filamentary nature.

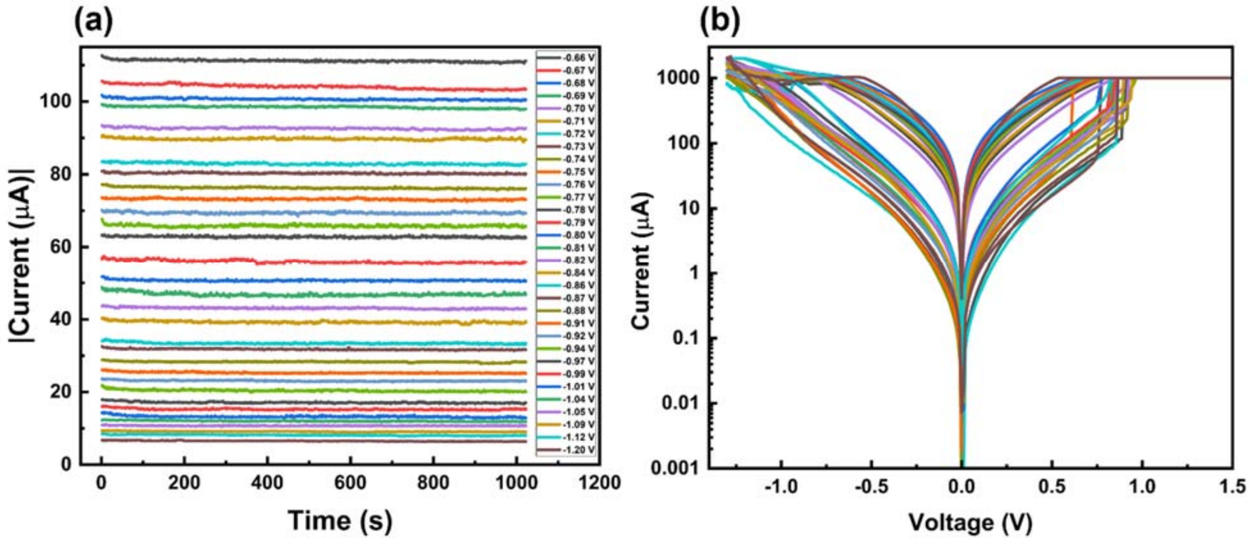

Figure S4. (a) Retention characteristics of the device after applying an incremental reset voltage sweep. With an increment of 0.01V we can set 32 states easily with a margin of approximate 0.5 μA. (b) Twenty Li-imbued TiO_x devices IV characteristics, showing the device-to-device variations.

5. Voice Recognition using Volatile and Nonvolatile Characteristics

Figure S5 represents the Cochleagram of voice data using Lyons passive air model and the binarized then reservoir computing (RC) processed data of a typical channel. **Figure S5a** shows the original voice data along with the binarized data (with a threshold value) and the RC processed data using the volatile properties of the devices. The x-axis is the time steps of the voice whereas y-axis represents the index number of the frequency channels, which are within human audible ranges. The pixels are the vector values, which are then binarized with a certain threshold and plotted in the middle of **Figure S5a**. Clearly, the binary values reduce the accuracy. When the binarized values are fed into the reservoir layers of volatile memory, the RC processed data better emulate the original voice data as shown on the right. **Figure S5b** shows a sample input of channel#30 and its corresponding current change. The conductance is potentiated up when the pulses are continuous and self-relaxes when there are no excitations. The conductance value increases again when the excitation resumes. The volatile property helps to add details to the binarized cochleagram.

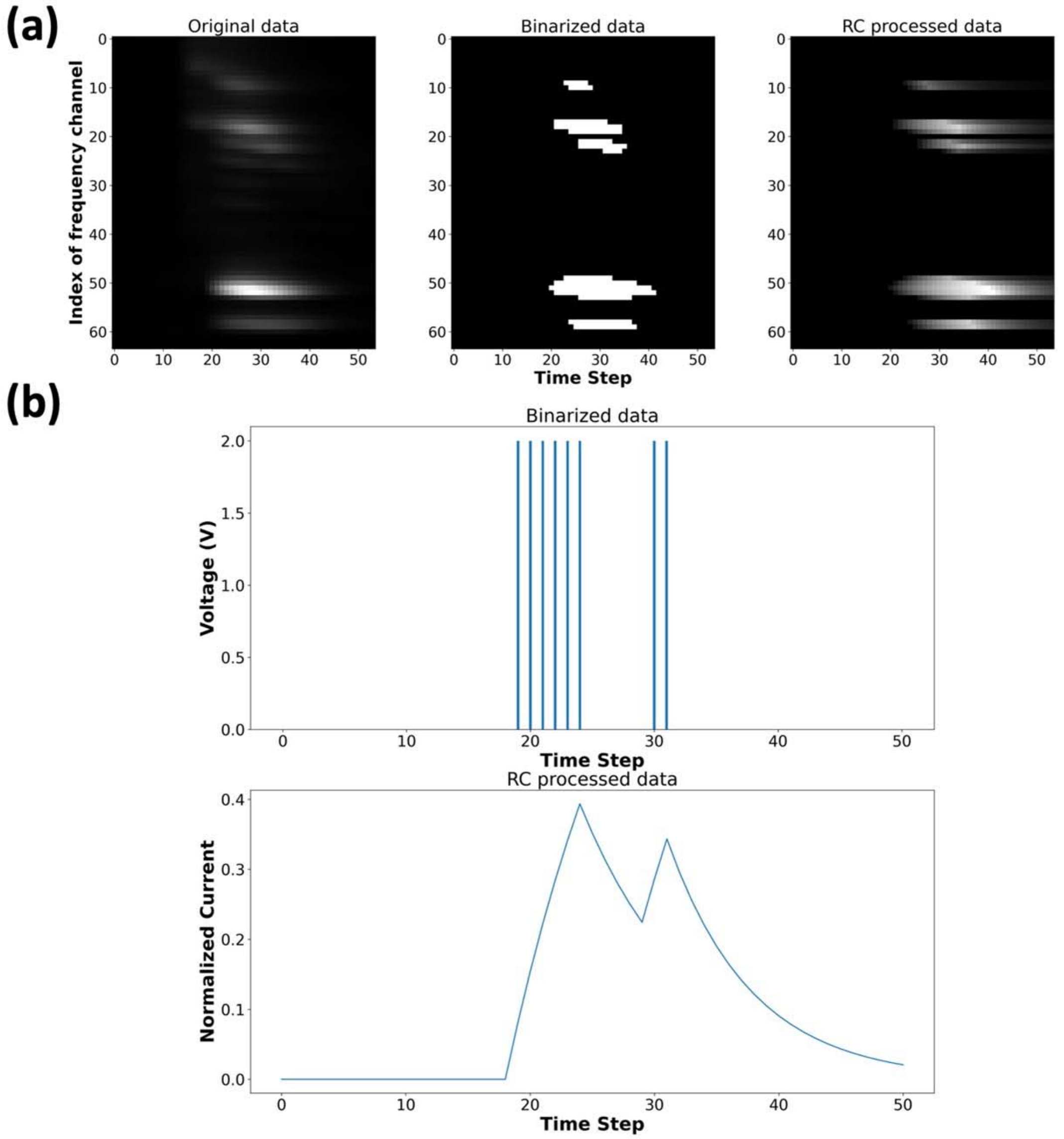

Figure S5. (a) Cochleagram of voice data, binarized data, and RC processed data. (b) Voltage input of channel #30 and corresponding current of volatile device at time scale.

6. Comparison with existing literature

Table S1 represents the comparison of key performance features of our demonstrated devices with existing literature. Experimentally determined variable time scale as shown in **Figure S2c** was used in our simulation. If we also use the conventional constant time scale assumption, the simulated accuracy can well exceed 97% for this particular task. Li-imbued TiO_x devices show tunable volatility and multi-bit nonvolatile capability, which are not present in other devices.

Device	Volatility	Volatile Time Tunability	Nonvolatility	Multi-bit Capability	Accuracy	Ref.
Pd/SiO_2:Ag/Pt	Yes	No	No	No	83%	(1)
Pd/Au/WO_x/W	Yes	No	No	No	99.2%	(2)
Ti/(TiO_x/TaO_y)/Pt	Yes	No	No	No	99.6%	(3)
Pt/ SiO_2:Ag/Pt	Yes	Yes	No	No	99.6%	(4)
TiN/TiO_x/LiPON/TiN	Yes	Yes	Yes	Yes	94.4%	[This Work]

Table S1. Performance parameter comparison between the existing literature and proposed device structure.

References:

(1) Midya, R.; Wang, Z.; Asapu, S.; Zhang, X.; Rao, M.; Song, W.; Zhuo, Y.; Upadhyay, N.; Xia, Q.; Yang, J. J. Reservoir computing using diffusive memristors. Advanced Intelligent Systems **2019**, 1, 1900084.

(2) Moon, J.; Ma, W.; Shin, J. H.; Cai, F.; Du, C.; Lee, S. H.; Lu, W. D. Temporal data classification and forecasting using a memristor-based reservoir computing system. Nature Electronics **2019**, 2, 480–487.

(3) Zhong, Y.; Tang, J.; Li, X.; Gao, B.; Qian, H.; Wu, H. Dynamic memristor-based reservoir computing for high-efficiency temporal signal processing. Nature communications **2021**, 12, 408.

(4) Ruiyi L.; Yang, H.; Zhang, Y.; Tang, N.; Chen, R.; Zhou, Z.; Liu, L.; Kang, J.; and Huang, P. Adjustable short-term memory of SiOx: Ag-based memristor for reservoir computing. Nanotechnology **2023**, 34, 50, 505207.
